# Nasopharyngeal carriage and antimicrobial susceptibility of *Haemophilus influenzae* among children younger than 5 years of age in Beijing, China

**DOI:** 10.1186/s12866-015-0350-7

**Published:** 2015-02-04

**Authors:** Hongbin Zhu, Aihua Wang, Jingjing Tong, Lin Yuan, Wei Gao, Wei Shi, Sangjie Yu, Kaihu Yao, Yonghong Yang

**Affiliations:** Key Laboratory of Major Diseases in Children and National Key Discipline of Pediatrics, Ministry of Education, National Clinical Research Center for Respiratory Diseases, Beijing Key Laboratory of Pediatric Respiratory Infection Diseases, Beijing Pediatric Research Institute, Beijing Children’s Hospital, Capital Medical University, Nan Li Shi Road 56, Beijing, 100045 China; Department of Pediatrics, The Second Hospital of Lanzhou University, Lanzhou, 730030 China; Department of Pediatrics, Beijing Chao-Yang Hospital Affiliated to Capital Medical University, Beijing, 100020 China

**Keywords:** *Haemophilus influenzae*, Antimicrobial susceptibility, Acute upper respiratory tract infection, Pediatrics

## Abstract

**Background:**

*Haemophilus influenzae* is one of the main pathogens that cause community-acquired respiratory infections in children. Our previous study showed that *H. influenzae* is the second most common pathogen causing pneumonia and accounts for 30–50% of bacterial meningitis among Chinese children. *H. influenzae* carriage in children and its resistance to commonly used antimicrobials varies widely both geographically and over time.

**Results:**

Surveys of the nasopharyngeal carriage of *H. influenzae* in children younger than 5 years of age with acute respiratory tract infection (ARI) were conducted in Beijing Children’s Hospital, China in 2000, 2002, 2010, and 2012. The overall annual carriage rates of *H. influenzae* among children younger than 5 years of age with ARI were 35.5%, 20.6%, 14.4%, and 18.7%, and the percentages of *H. influenzae* isolates producing β-lactamase were 4%, 13%, 27.1%, and 31%, respectively. The percentages of susceptibility to ampicillin progressively decreased from 96% (2000) to 87% (2002) to 63% (2010) to 61% (2012). All of the ampicillin-resistant isolates were found to be beta-lactamase producers. The susceptibility to tetracycline increased from 54% (2000) to 60% (2002) to 91.5% (2010) to 94.5% (2012). No statistically significant differences were observed in the susceptibility to cefaclor, cefuroxime, sulfamethoxazole, and chloramphenicol. Amoxicillin/clavulanic acid and ceftriaxone were the most effective antimicrobials for the isolates of *H. influenzae* across the 10-year period.

**Conclusions:**

This report on the *H. influenzae* carriage rates in children and the susceptibility of these bacteria to commonly used antibiotics showed that *H. influenzae* carriage decreased from 2000 to 2012. Additionally, the percentage of β-lactamase-producing isolates increased while their susceptibility to ampicillin progressively decreased during this time. These results indicate that the appropriate empirical antimicrobial therapy should be changed for pediatric patients in China.

## Background

*Haemophilus influenzae* is a Gram-negative bacteria commonly found in the upper respiratory tract of healthy children and adults and is one of the main pathogens that cause community-acquired respiratory tract infections during childhood [1]. Prior to the introduction of Type b *H. influenzae* (Hib) vaccines, Hib was thought to be responsible for approximately three million serious illnesses and an estimated 386,000 deaths per year, with 95% of cases and 98% of deaths worldwide occurring among patients in developing countries [2]. The primary diseases caused by *H. influenzae* are childhood pneumonia, meningitis, and bacteremia. Almost all victims are children under 5 years of age, with those between 4 and 18 months of age being especially vulnerable. Our previous studies have shown that the overall nasopharyngeal carriage rate of *H. influenzae* is 26.3% among children younger than 5 years old with acute respiratory tract infection (ARI). Meanwhile, *H. influenzae* meningitis accounts for 30–50% of bacterial meningitis, and Hib is the second most common pathogen causing pneumonia in Chinese children [3,4].

The resistance of *H. influenzae* to commonly used antimicrobials has risen in recent years, and wide variations exist in antimicrobial susceptibility rates, both geographically and over time [5]. Previous reports have demonstrated that pneumococcal and Hib conjugate vaccines can prevent nasopharyngeal acquisition of pediatric serotypes in children and subsequent transmission to others. During our study, both the pneumococcal and the Hib conjugate vaccines were available to the private sector in Beijing, but most children did not receive these vaccines. Investigating the geographical variations in antimicrobial resistance and monitoring trends in antimicrobial resistance development are essential for adequate antibacterial therapy. The present study investigates and compares the antimicrobial susceptibility of *H. influenzae* strains isolated from children younger than 5 years of age with ARI at Beijing Children’s Hospital in China from 2000 to 2002 and from 2010 to 2012.

## Methods

### Study population

Beijing Children’s Hospital is the largest general children’s hospital in China, with over 700 beds and around 6,000 child visits per day. During the study period, outpatients younger than 5 years of age displaying symptoms of ARI were enrolled in this study. Nasopharyngeal samples were collected from children with ARI during February through May for each of the study years, and the subjects received neither vaccines nor antibiotics in the 15 days prior to the nasopharyngeal sample collection. The project was approved by the Ethics Committee of Beijing Children’s Hospital, and consent was obtained from the parents or guardians of the enrolled children.

### Microbiological methods

Nasopharyngeal samples were collected using cotton-tipped swabs, which were immediately placed in a transfer cultivation medium comprised of degreased milk, glucose, and glycerin, as recommended by the Center for Disease Control and Prevention, USA. The samples were sent to the hospital’s microbiological laboratory within 2 hours for monoculture of *H. influenzae*. Cultures were grown in chocolate medium dishes incubated at 37°C in a 5% CO_2_ incubator and were examined after 24–48 hours. Bacterial identification was carried out following standard procedures using colony morphologic features, Gram staining, and a requirement for both X and V factors (Oxoid, Basingstoke, UK). Detection of β-lactamase activity in the clinical isolates was determined by the chromogenic cephalosporin nitrocefin (BR66A; Oxoid) method using known β-lactamase positives as controls.

Antibiotic susceptibility tests for *H. influenzae* isolated from each patient at each collection period in 2000 and 2002 were performed according to the National Committee for Clinical Laboratory Standards (NCCLS) guidelines [6] and the instructions from the manufacturer of the antibiotics (AB Biodisk). Quality control was carried out by using *H. influenzae* ATCC 10211, ATCC 49247, and ATCC 49766. Antibiotic susceptibility to ampicillin (AMP), amoxicillin/clavulanic acid (AMC), cefaclor (CFC), cefuroxime (CXM), and ceftriaxone (CRO) were determined through E-tests (AB Biodisk, Solna, Sweden). MIC_50_ and MIC_90_ were defined as the minimal concentration of a given antibiotic agent needed to inhibit 50% and 90% of the growth of the isolates, respectively. Tetracycline (TCY), sulphamethoxazole (SXT), and chloramphenicol (CHL) susceptibilities were detected using the Kirby–Bauer disk diffusion method (Oxiod, Basingstoke, Hampshire, England).

In 2010 and 2012, antibiotic susceptibilities of the clinical isolates of *H. influenzae* were determined using the broth microdilution method on haemophilus test medium (HTM) method, according to the Clinical and Laboratory Standards Institute (CLSI) guidelines [7]. The antimicrobial susceptibilities to AMP, AMC, CFC, CXM, CRO, TCY, SXT, and CHL were tested.

### Data analysis

The carriage rates of *H. influenzae* were evaluated by annual percentages, and the antibiotic sensitivities were expressed as the number of susceptible isolates/total number of tested isolates. A chi-square test was performed to compare temporal susceptibility for *H. influenzae* using the statistical software SPSS 16.0 for Windows, and the two-tailed cutoff of *p* < 0.05 was considered statistically significant.

## Results

### Carriage rates and β-Lactamase production of *H. influenzae*

The annual percentages of *H. influenzae* carriage and of *H. influenzae* isolates producing β-Lactamase during the study years are listed in Table 1. A total of 204 and 144 isolates were identified from the nasopharyngeal specimens among 782 and 882 children with ARI, respectively, during the two study periods. The carriage rates of *H. influenzae* among the children in the two study periods were statistically different from one another (*p* < 0.01). The overall carriage rate of *H. influenzae* among children with ARI decreased from 26.1% in 2000 and 2002 to 16.3% in 2010 and 2012, whereas the percentage of β-lactamase-producing isolates increased from 4%–13% to 27.1%–31% over the span of 10 years.

**Table 1:** **Carrier rates and prevalence of β-lactamase production in** ***H. influenzae*** **isolates among children younger than five years old with ARI**

**Years**	**No. of cases**	**No. of isolates (%)**	**β-lactamase production (%)** ^**1**^
2000	292	103 (35.5)	4/100 (4.0)
2002	490	101 (20.6)	13/100 (13.0)
Total 2000 and 2002	782	204 (26.1)	17/200 (8.5)
2010	487	70 (14.4)	19/70 (27.1)
2012	395	74 (18.7)	23/74 (31.0)
Total 2010 and 2012	882	144 (16.3)	42/144 (29.2)

^1^The numbers tested for β-lactamase were not same as the number of isolates in 2000 and 2002 because four strains died before being tested.

### *H. influenzae* antimicrobial susceptibilities

Table 2 shows the annual susceptibility results for the *H. influenzae* isolates to the eight antimicrobial agents tested. The prevalence of *H. influenzae* susceptibility to ampicillin progressively decreased from 2000 to 2002 and again in 2010. Meanwhile, no statistically significant trends were observed in the number of *H. influenzae* isolates that were susceptible to amoxicillin/clavulanic acid, cefaclor, cefuroxime, ceftriaxone, sulfamethoxazole, or chloramphenicol during the longitudinal study period (*p* > 0.05). The prevalence of *H. influenzae* susceptibility to tetracycline increased from 60% and 54% in 2000 and 2002 to 91.5% and 94.5% in 2010 and 2012. Over the two periods, 27 (in 2000 and 2002) and 42 (in 2010 and 2012) isolates resistant to ampicillin were identified. All of these ampicillin-resistant isolates were β-lactamase producers, and no β-lactamase-negative ampicillin-resistant strains were identified. The production of β-lactamase did not affect the susceptibility of the isolates to amoxicillin/clavulanic acid, and throughout the 10-year period the *H. influenzae* isolates displayed the highest sensitivities to both amoxicillin/clavulanic acid and ceftriaxone.

**Table 2:** ***In vitro*** **activity (MIC**_50_ **, MIC**_90_ **, and MIC range, μg/L) and percentage of** ***H. influenzae*** **isolates that were susceptible (S), intermediate (I), or resistant (R) to antibiotic agents during the study period**

**Antibiotics agents**	**Year**	**MIC**_**50**_	**MIC**_**90**_	**MIC range**	**S (%)**	**I (%)**	**R (%)**
Ampicillin (AMP)	2000	0.19	0.44	0.064–256	96	0	4.0
	2002	0.25	1	0.064–256	87	0	13.0
	2010	0.75	256	0.064–256	63.0	9.9	27.1
	2012	0.75	256	0.064–256	61.2	7.8	31.0
Amoxicillin / clavulanic acid (AMC)	2000	0.5	0.75	0.19–3	100	0	0
	2002	0.5	1	0.064–4	100	0	0
	2010	0. 5	2	0. 19–3	100	0	0
	2012	0.75	3	0. 19–8	97.8	0	2.7
Cefaclor (CFC)	2000	1.5	2	0.5–48	98.0	1.0	1.0
	2002	1	2	0.38–48	95.0	2.0	3.0
	2010	1.5	16	0.5–128	87.2	5.7	7.1
	2012	1.5	12	0.5–256	81.0	6.8	12.2
Cefuroxime (CXM)	2000	0.5	0.75	0.19–4	100	0	0
	2002	-	-	-	99	1.0	0
	2010	0.75	1	0.75–4	97.1	2.9	0
	2012	0.75	1	0.75–4	91.9	2.7	5.4
Ceftriaxone (CRO)	2000	0.004	0.006	0.002–0.032	100	0	0
	2002	-	-		99	1.0	0
	2010	0.008	0.016	-	100	0	0
	2012	0.016	0.047	0.004–0.032			
0.016–0.75	100	0	0				
Tetracycline (TCY)	2000	-	-	-	60.0	23.0	17.0
	2002	-	-	-	54.0	24.0	22.0
	2010	-	-	-	91.5	7.1	1.4
	2012	-	-	-	94.5	4.1	1.4
Sulfamethoxazole (SXT)	2000	-	-	-	21.0	0	79.0
	2002	-	-	-	37.0	1.0	62.0
	2010	-	-	-	22.9	1.4	75.7
	2012	-	-	-	28.4	2.7	68.9
Chloramphenicol (CHL)	2000	-	-	-	93.0	1.0	6.0
	2002	-	-	-	87.0	1.0	12.0
	2010	-	-	-	88.6	11.4	0
	2012	-	-	-	95.9	0	4.1

## Discussion

The human nasopharynx is an important reservoir and vector, and bacteria carried here can cause community-acquired pneumonia as well as some invasive infections. The prevalence of nasopharyngeal carriage of potential pathogens varies based on age, geographical area, crowding, concomitant respiratory tract illness, antibiotic consumption, sampling technique, and type of infant feeding [8]. Seasonal variations occur in the prevalence of nasopharyngeal carriage of respiratory pathogens in children, but the differences have been slight and had limited clinical relevance [9]. One study from India indicated that nasal carriage rates of *H. influenzae* in male and female child populations were significantly different from one another [10]. In Spain, the average bacterial carriage rate was 42% in healthy children attending day care centers, but the rates were highly variable between centers (range: 12% to 83%). When samples from these children were analyzed, 99% of the bacterial isolates were identified as non-typeable *H. influenzae* [11].

The present study indicates that in Beijing the overall carriage of nasopharyngeal *H. influenzae* among children younger than 5 years of age with ARI decreased from 26.8% in 2000 and 2002 to 16.3% in 2010 and 2012, while the percentage of β-lactamase-producing isolates increased from 8.5% to 29.2% over the study period. This result is similar to that observed in Northern Taiwan, where the rate of nasopharyngeal *H. influenzae* carriage among healthy children younger than 5 years was low (5.6%), and the prevalence of β-lactamase producers was up to 61.5% [12]. The results from the early years of our current study (2000 and 2002) were in line with those from our previous study, which showed that in 2000 to 2004 the rate of *H. influenzae* nasopharyngeal carriage in a similar child population was 26.3%, and 10.9% of the isolated strains produced β-lactamase [4]. The percentage of *H. influenzae* strains producing β-lactamase in the present study differed from the reported percentages among Chinese adults, especially for the more recent years, in which 9.5% in 2002 to 2003 and 13.1% in 2009 to 2010 of *H. influenzae* produced β-lactamases [13,14].

Notably, the rates of *H. influenzae* carriage in the child populations from this study decreased from 26.1% to 16.3% in the span of 10 years. The nasopharynx of a child may represent a unique dynamic environment modulated by intricate interactions between bacterial species, host immune system, and vaccine immunization [15]. Nasopharynx flora composition is influenced not only by age but also by socio-economic setting. Presumably, many factors contribute to our findings, including individual patterns of using antibiotics and immunization of some children with the Hib vaccine during the study period.

*H. influenzae* resistance to ampicillin emerged in the early 1970s and has increased steadily since then. The production of β-lactamase is the most common mechanism of resistance to β-lactam antibiotics, such as ampicillin, with β-lactamase-negative ampicillin-resistant (BLNAR) strains being relatively rare. The present study found that the rates of β-lactamase-producing *H. influenzae* isolates increased from 4% in 2000 to 13.0% in 2002. These percentages are similar to the frequencies with which β-lactamase-producers were isolated in Japan and the Czech Republic around the same time, where the percentages of β-lactamase-producing *H. influenzae* were 5.2% (2000–2001) and 8.0% (2002–2003) in Japan and 4.6% (2004–2005) in the Czech Republic [16,17]. The present study also demonstrated that the percentage of β-lactamase-producing isolates increased to 27.1% in 2010 and to 31% in 2012. Similarly, one surveillance study of 1,545 strains from 2008 to 2010 in the United States revealed an overall prevalence of 26.7% for β-lactamase-positive strains, ranging from 17.9% to 38% across different age groups [18]. The increased rates of β-lactamase-positive strains and resistance to ampicillin may be caused by the extensive use of β-lactam antibiotics in the United States. Meanwhile, penicillins and cephalosporins are the most commonly prescribed antibiotic agents in pediatric population in China [4].

The present study indicated that the susceptibility of *H. influenzae* to ampicillin decreased from 87%–96% to 61.2%–63% over the last decade. Colonized bacteria are frequently exposed to antibiotics, particularly in regions susceptible to the disease. This can lead to the selection of antibiotic-resistant bacteria that can be subsequently transmitted and cause invasive diseases. Antibiotic utilization is one of the documented risk factors for the development of antimicrobial resistance. Previously, 98% of the pediatric outpatients with common colds were prescribed with antibiotic agents in Beijing Children’s Hospital in the 1990s [19], and the total usage of antibiotics among outpatients was approximately 40 defined daily doses/100 patient days from 2000 to 2004 [4]. In China, amoxicillin/clavulanic acid and cephalosporins were the most commonly prescribed antibiotics for pediatric outpatients over the last two decades. In addition, cefaclor and oral cefuroxime could be easily obtained from various clinics or drug stores. Likely because of this high usage, the resistance to amoxicillin/clavulanic acid emerged in 2012, and the susceptibility of *H. influenzae* to cefaclor and cefuroxime decreased during the study period.

Third-generation cephalosporins exhibited the highest activity against *H. influenzae* strains isolated from the nasopharyngeal samples in this child population during the monitored 10-year interval. Thus, ceftriaxone had the lowest MIC_50_ and MIC_90_ values, as well as the highest proportion (99% to 100%) of *H. influenzae* that were susceptible to ceftriaxone. The high activity of ceftriaxone against *H. influenzae* isolates in children is similar to that observed in the United States from 2005 to 2007 [20].

Tetracycline resistance of *H. influenzae* is associated with an efflux mechanism encoded by the *tet* (B) gene, which is usually located on conjugate plasmids. The present study indicated that the rates of susceptibility to tetracycline of *H. influenzae* isolated from children increased from 54%–60% to 91.5%–94.5% over the 10-year period. This increase in tetracycline-susceptible strains of *H. influenzae* may be related to the decrease in tetracycline usage because this antibiotic was not used in most areas of China for more than 20 years. Trimethoprim and sulfamethoxazole (used alone or in combination) provide an antimicrobial effect by interfering with the metabolism and replication of bacteria through a sequential blocking of tetrahydrofolate production. Resistance to trimethoprim-sulfamethoxazole among strains of *H. influenzae* is common and is caused by an increase in the production of dihydrofolate reductase (DHFR) with altered affinity for trimethoprim [21]. The prevalence of susceptibility to sulfamethoxazole ranged from 62% to 79% over the 10-year period.

The rates of *H. influenzae* resistance to chloramphenicol remained stable over the 10-year period. This phenomenon may be associated with the decrease in chloramphenicol usage in China, where this antibiotic agent has been prescribed only rarely for infectious diseases in the last three decades, especially in children. Chloramphenicol had not been used in Beijing Children’s Hospital since 1999. However, not surprisingly, the frequency of chloramphenicol-resistant *H. influenzae* progressively increased from 1994 to 2002 in Kenya, Africa, where chloramphenicol was the main antibiotic administered for *H. influenzae* infections among children older than 2 months [22]. *H. influenzae* resistance to chloramphenicol is usually associated with a plasmid-mediated production of chloramphenicol acetyltransferase (CAT), encoded by the *cat* gene, with occasional strains having a penetration barrier. Furthermore, the plasmid often carries genes encoding resistance to tetracycline and ampicillin [23]. However, the combination of resistance to these three antibiotics in the *H. influenzae* strains isolated from children in this study was not tested.

*H. influenzae* is one of the principal community-acquired respiratory tract bacterial pathogens. Continued epidemiological monitoring of the nasopharyngeal carriage and the susceptibility status of these strains is required. The present study provided nasopharyngeal colonization data on the important characteristics of *H. influenzae* strains prevalent in the child population in China. This study also determined the status of *H. influenzae* resistance to several different antibiotics, which is helpful for practicing appropriate empirical antimicrobial therapy in pediatric patients.

## Conclusion

The overall carriage rates of *H. influenzae* among children younger than 5 years of age with ARI were decreased from 26.1% in 2000 and 2002 to 16.3% in 2010 and 2012, while the percentage of β-lactamase-producing isolates increased from 4%–13% (2000–2002) to 27.1%–31% (2010–2012) over the span of 10 years. All of the isolates found to be resistant to ampicillin were β-lactamase producers, and the production of β-lactamase did not affect the susceptibility of the isolates to amoxicillin/clavulanic acid. Additionally, ceftriaxone was the most effective antimicrobial agent against the isolates of *H. influenzae* over the two study periods. Our results indicate that the appropriate empirical antimicrobial therapy should be changed for pediatric patients in China.
